# Comparison of percutaneous curved kyphoplasty and bilateral percutaneous kyphoplasty in osteoporotic vertebral compression fractures: a randomized controlled trial

**DOI:** 10.1186/s12891-021-04469-1

**Published:** 2021-06-26

**Authors:** Chen Wang, Yu Zhang, Wang Chen, Shi-Lei Yan, Kai-Jin Guo, Shuo Feng

**Affiliations:** 1 Department of Orthopedic Surgery, Nanjing Medical University, Nanjing, Jiangsu China; 2 Department of Orthopedic Surgery, Affiliated Hospital of Xuzhou Medical University, 99 Huaihai Road, Xuzhou, 221002 Jiangsu China

**Keywords:** Osteoporosis, Vertebral compression fractures, Percutaneous curved kyphoplasty, Kyphoplasty

## Abstract

**Background:**

Here we compared the clinical efficacy of bilateral percutaneous kyphoplasty (PKP) and percutaneous curved kyphoplasty (PCKP) in the treatment of osteoporotic vertebral compression fractures (OVCF).

**Methods:**

Seventy-two patients with single-level thoracolumbar OVCF were randomly divided into 2 groups (36 patients in each) and were subjected to either PCKP or bilateral PKP. The intraoperative fluoroscopy time, total surgical time, bone cement injection volume, bone cement leakage, preoperative and postoperative anterior vertebral height, Cobb angles, visual analog scales (VAS) and oswestry disability index questionnaire (ODI) were recorded.

**Results:**

Both groups of patients had a trend towards improvements in VAS and ODI scores 24 h and 6 months after surgery, when compared to preoperative results, despite lack of statistical significance. The total surgical and intraoperative fluoroscopy times and intraoperative bone cement injection volume were significantly decreased in the PCKP group than those in the PKP group. The anterior edge height and Cobb angle of the injured vertebra were similarly improved after operation in both groups.

**Conclusion:**

PCKP is safer, less invasive and quicker than traditional bilateral PKP despite similar short-term effects for the treatment of OVCF.

**Trial registration:**

ChiCTR, ChiCTR2100042859. Registered 25 January 2021- Retrospectively registered.

## Background

Osteoporotic vertebral compression fractures (OVCF) [1] are common clinical fractures, especially in the elderly. The loss of bone height and vertebral body compression fractures lead to kyphotic deformities in the vertebral spine of patients, causing long-term back pain and seriously affecting their quality of life. A conservative approach using pharmacological treatment is appropriate for specific populations [2].

At present, percutaneous kyphoplasty (PKP) is widely performed clinically to treat OVCF [3–5]. Balloon expansion can be used to correct kyphoplasty and make a cavity, so that bone cement can be injected at a relatively low pressure to reduce leakage, and postoperative pain can be significantly relieved. The commonly used puncture approaches include a unilateral straight approach and a bilateral transpedicular approach, both of which can improve the back pain of patients [3, 5]. However, in the traditional unilateral straight approach, bone cement can often only fill the ipsilateral vertebral bone, leaving the contralateral vertebral bone poorly filled [5]. The bilateral transpedicular approach also has disadvantages, such as a long operation time, need of several punctures and a large amount of radiation, which can lead to the “binocular” phenomenon that occurs when the bone cement injected from both sides do not fuse [3]. Therefore we have focused our research on achieving a uniform distribution of bone cement in the vertebral body through a unilateral approach. In recent years, the introduction of percutaneous curved kyphoplasty (PCKP) provided a new approach for the treatment of OVCF [6].

Thus, the present prospective study compared the clinical efficacy of PCKP with that of PKP in OVCF to define the optimal surgical treatment.

## Methods

### Study design

**Fig. 1:**
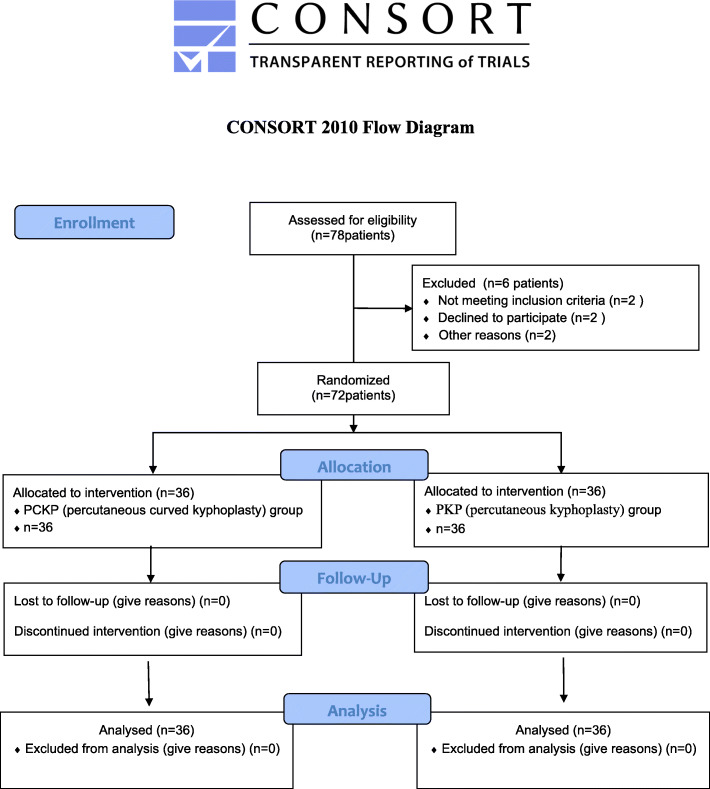
Flow chart of the analysis. After exclusions, a total of 72 patients were followed up

This is a prospective and controlled study that included researchers who analyzed patient data and were trained in the study methods but did not know the patient population. This study was conducted in our orthopedic Department from January 2019 to January 2020. This study was approved by the ethics Committee of our hospital, and its design conforms to the Regulations on the Management of Medical Institutions. This trial is registered at Chinese Clinical Trials Registry, number ChiCTR2100042859. The CONSORT flow diagram is provided in Fig. 1.

### Inclusion and exclusion criteria

The inclusion criteria for this study were patients with OVCF who were able to tolerate surgery and cooperate with postoperative functional exercise in our hospital. Patients in the study were informed of treatment plans and surgical risks and signed informed consent forms. Therefore, the inclusion criteria were: (1) patients aged > 65 years, both men and women; (2) painful OVCF and (3) granted informed consent to enroll in the trial.

The exclusion criteria were (1) vertebra tuberculosis and bacterial infection; (2) bleeding and clotting dysfunctions that could not be corrected or patients with a bleeding tendency; (3) extensive and largely incomplete vertebral posterior margin bone destruction; (4) > 70% compression degree of vertebral bodies; (5) two or more vertebral bodies compression fractures; (6) participation in other drug or medical device clinical trials within 30 days prior to screening and (7) the researcher judged that the patient had poor compliance and could not complete the study as required.

### Patient randomization and sample size calculation

A total of 78 patients were initially identified. Two patients did not meet inclusion criteria, 2 patients refused to participate, 2 patients were lost during follow-up, leaving a total of 72 patients included. There were 22 males and 50 females, aged 66–87 years (average, 76.04 years). Preoperative routine examination consisted of blood routine (Hb, HBC, WBC and lymphocyte count), coagulation routine (TT, APTT and PT) and lower limb color Doppler ultrasonography. At the time of admission, patients were numbered consecutively from 1 to 72. The patients were randomly divided into a study group (*n* = 36) and a control group (*n* = 36) using a random distribution software. The selection process of patients in the two groups is shown in Fig. 1.

To calculate the sample size, the observational cohort study was powered to detect bone cement infusion volume as the minimum mean difference of significance, and calculated standardized difference (0.953) using standard deviation (1.202) based on an earlier report by Cheng et al. [6]. It was estimated that 32 participants would be required to enable detection of significant differences, at the 5% significance level, with 95% power.

### Surgical procedures

#### PCKP (percutaneous curved kyphoplasty) group

**Fig. 2:**
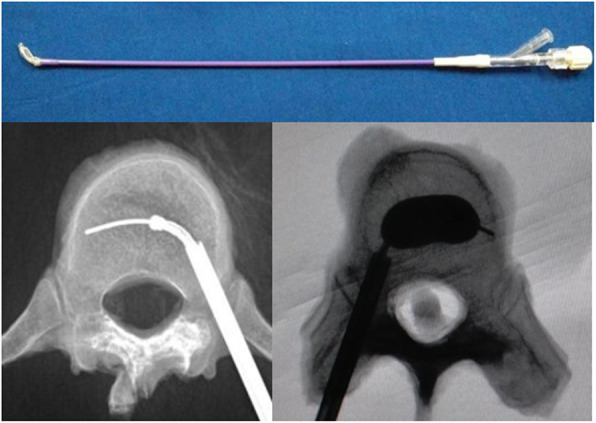
Percutaneous curved kyphoplasty puncture needle (Ningbo Huarun Biotechnology Co., LTD., China)

Percutaneous curved kyphoplasty puncture needle and bone cement high-pressure perfusion instrument were purchased from Ningbo Huarun Biotechnology Co. (China), while acrylic resin bone cement was purchased from StrykerInstruments (France) (Fig. 2).

**Fig. 3:**
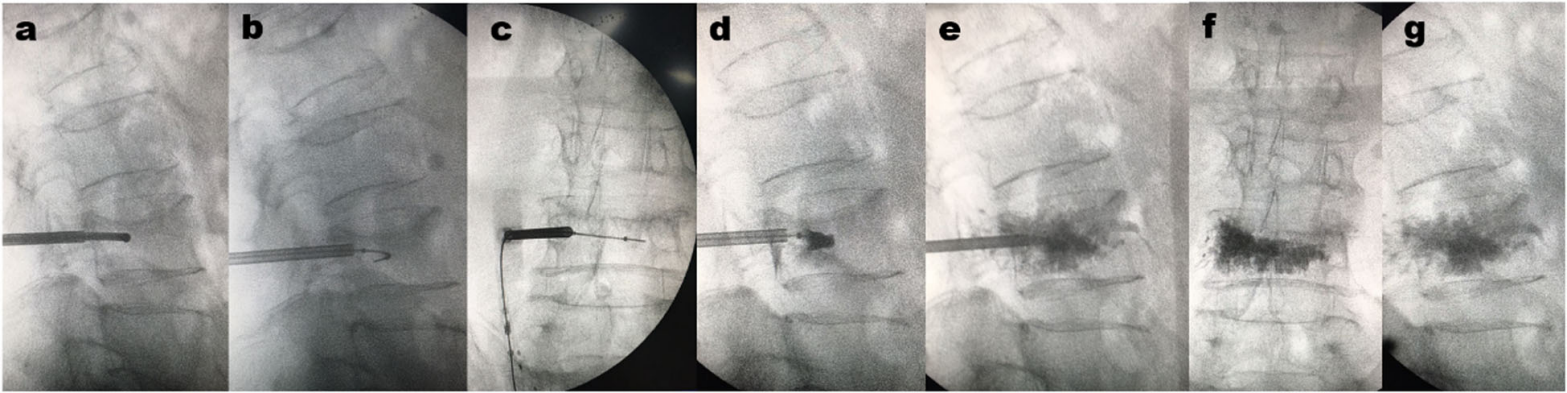
Representative intraoperative fluoroscopy imaging data obtained during percutaneous curved vertebroplasty

The patient was placed in a prone position, with a soft padded chest. The C-arm machine was used for fluoroscopy, to locate the damaged vertebral body and to determine and mark the pedicle puncture needlepoint. After local anesthesia, a unilateral pedicle puncture was performed, and the puncture needle was placed at the posterior 1/3 of the vertebral body. After the core of the puncture needle was pulled out, the curved catheter was placed into the vertebra along the straight cannula. The anteroposterior fluoroscopy showed that the end of the curved catheter crossed the midline of the vertebra to reach the opposite side, while the lateral fluoroscopy reached 1/3 of the anterior middle vertebra. The working channel at the bending angle of the vertebral body was established. The vacuum-extracted vertebral dilation balloon catheter was inserted into the vertebral body through the working channel. The balloon had completely entered the vertebral body when the proximal black mark entered the puncture channel, upon which it was expanded and the expected position of the vertebral body was observed through fluoroscopy. The bone cement was mixed evenly and injected into the established expansion channel through the injection delivery system. Then the bone cement was slowly injected into the vertebral body. At the same time, the patient’s vital signs were closely monitored. When the bone cement was evenly distributed and approached the posterior wall of the vertebral body the injection was stopped. After the injection was completed and the bone cement outside the body was completely hardened, needle was rotated and pulled out the sleeve to prevent the tailing phenomenon (Fig. 3).

#### PKP (percutaneous kyphoplasty) group

PKP was performed with a bilateral approach using PKP apparatus and bone cement from Kyphon Corporation (USA). Preoperative localization, disinfection and anesthesia were identical to PCKP. C-arm fluoroscopy guided down simultaneous punctures of the bilateral pedicles, then the expanded trocar was successively placed, fine drilled and balloon expanded at the collapsed position in front of the vertebral body. After the balloon dilation was sufficient, bone cement was prepared and injected into the diseased vertebral body at the wiredrawing stage. The injection stopped when the bone cement was found to satisfactorily fill the vertebra, being distributed to the edge of the vertebral body or spilled out of the vertebral body under fluoroscopy.

### Clinical and radiographic assessment

The duration of surgery, C-arm X-ray frequency, bone cement injection volume, bone cement leakage rate, anterior edge height of diseased vertebra before and after surgery and kyphosis Cobb angle of the vertebra were recorded. The visual analog scale (VAS) and functional disturbance index (Oswestry disability index, ODI) were recorded before surgery and 24 h and 6 months after surgery, in order to assess the curative effect of surgeries.

The bone cement injection volume in the PCKP group was calculated according to the scale on the puncture trocar, while for the PKP group this was calculated as 1.5 mL × the number of pipes. The bone cement leakage rate was assessed by conventional X-ray 1 day after the surgery. Cobb’s angle and anterior height of diseased vertebra were measured preoperatively and 24 h and 6 months after surgery by lateral radiographs.

### Statistical analysis

The analysis and production of data and charts were processed by IBM SPSS Statistics 19.0 statistical software (IBM, Chicago, USA) and GraphPad Prism 6.0 (GraphPad Software, San Diego, USA). Continuous variables were analyzed using independent t-tests. Categorical variables were analyzed using the Pearson chi-square or Fisher exact tests. Test level was set at both sides α =0.05 and *P* < 0.05 was considered statistically significant.

## Result

### Basic surgical conditions

The total surgical and intraoperative fluoroscopy times of the PCKP group were significantly lower than those of the traditional PKP group (*P*<0.05, Table 1). In addition, there was a trend towards a different intraoperative bone cement leakage between the two groups, although there was no statistical difference, There were 3 cases of leakage in the PCKP group (1 case in the intervertebral space, 1 case in the lateral position of the diseased vertebrae, and 1 case in the anterior position of the diseased vertebrae), and 8 cases in the PKP group (3 cases in the intervertebral space, 1 case in the paravertebral segment intravascular, 2 cases in the lateral position of the diseased vertebrae, and 2 cases in the anterior position of the diseased vertebrae). Among the 72 cases, there were no serious complications such as pulmonary embolism, spinal canal stenosis, spinal cord compression or nerve injury 6 months after the operation.

**Table 1:** Comparison of basic data between the two groups

classification	PCKP group	PKP group	*P* values
Number(n)	36	36	
Sex (male/female)	10/26	12/24	0.609
Age (years)	75.55 ± 6.11	76.52 ± 6.24	0.506
BMI kg/m^2^)	22.99 ± 2.06	23.19 ± 1.97	0.674
BMD	2.55 ± 0.65	2.62 ± 0.78	0.684
Comorbidity			
Hypertension	9	11	0.599
Diabetes	7	6	0.759
operative time (min)	39.30 ± 7.87	48.19 ± 9.00	0.000
X-ray frequency (n)	19.97 ± 4.70	29.66 ± 5.98	0.000
Infusion volume (ml)	3.84 ± 0.55	4.78 ± 0.67	0.000
Cement leakage (n)	3	8	0.101

### Perfusion, leakage and distribution of bone cement

In the PCKP group, bone cement was injected from a single perfusion point, and the average perfusion volume was 3.84 ± 0.55 ml. However, after bilateral balloon dilation in the vertebral body, the space to accommodate cement was significantly increased in the traditional PKP group. Therefore, the traditional PKP group had a higher bone cement perfusion volume, with an average perfusion volume of 4.78 ± 0.67 ml, compared with that of the PCKP group (*P*<0.05, Table 1). In the traditional PKP group, there was only 1 case of small paravertebral segment intravascular leakage and no distant intravascular leakage. Three cases of bone cement leakage occurred in the PCKP group in the paravertebral body without vascular leakage. When the puncture needle reached the ideal position in the vertebral body, bone cement could be distributed in the anterior and middle part of the vertebral body in PCKP group (Fig. 3), while the bone cement injected by traditional PKP after bilateral balloon dilation was mainly distributed in the sides of the vertebral body.

### Radiographic results

**Fig. 4:**
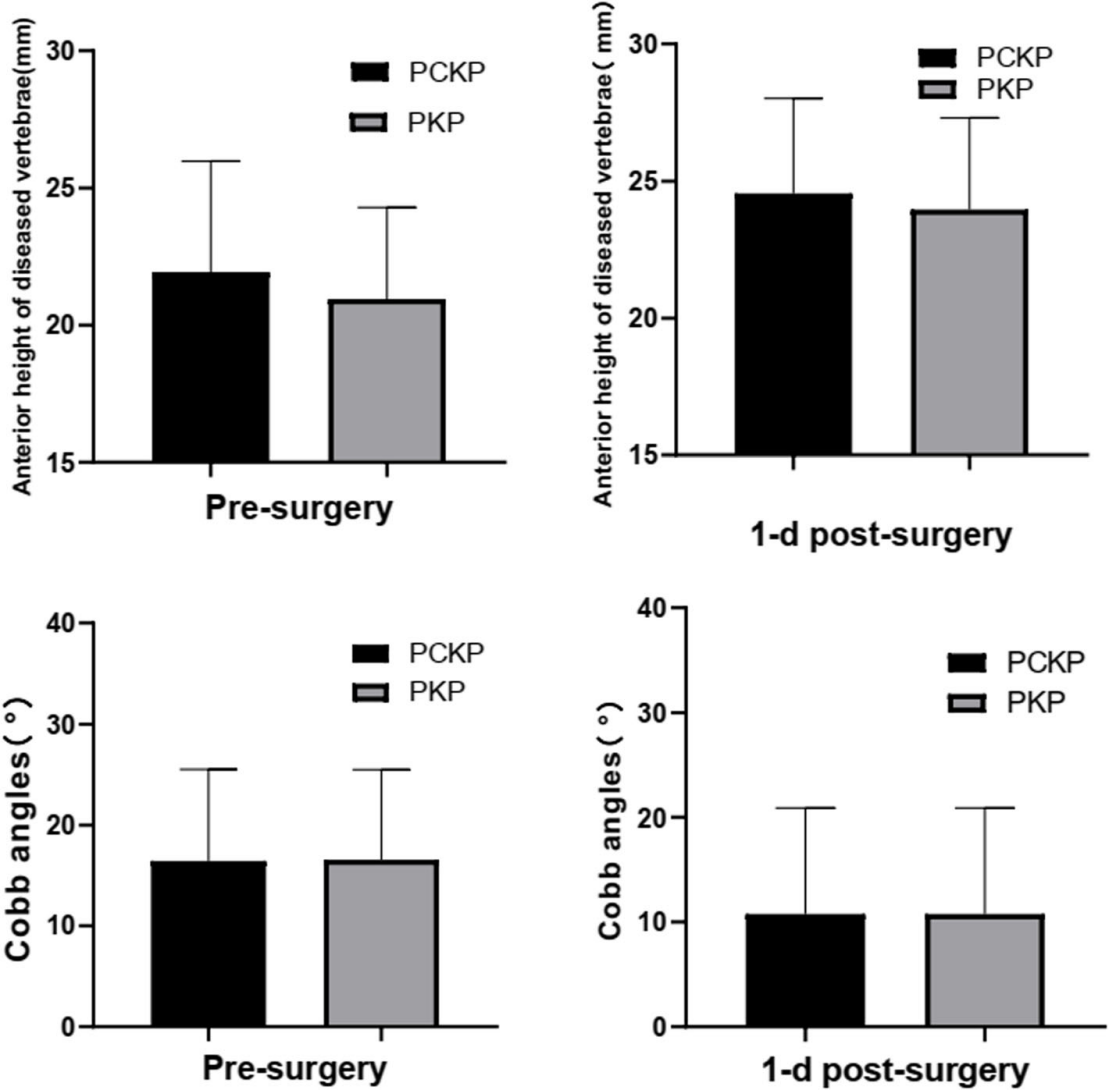
Comparison of the preoperative and postoperative anterior height of diseased vertebrae and cobb angles between the two groups

The anterior height of the vertebral body in the PCKP group and traditional PKP group were respectively 21.93 ± 4.05 mm and 20.95 ± 3.34 mm before the operation, and were increased by 2.79 mm (final 24.72 ± 3.47 mm) and 3.01 mm (final 23.96 ± 3.36 mm) respectively after the operation(*P*<0.05) (Fig. 4). However, there was no statistically significant difference in the height of the anterior vertebral body between the two groups before and after surgery (*P*>0.05, Table 2).

**Table 2:** Comparison of pain and functional efficacy between the two groups

classification	PCKP group	PKP group	*P* values
**VAS score (score)**			
Pre-surgery	7.58 ± 1.30(6 ~ 10)	7.39 ± 0.96 (6 ~ 10)	0.473
24 h post-surgery	2.11 ± 0.57 (1 ~ 3)	2.00 ± 0.58 (1 ~ 3)	0.419
6-months post-surgery	1.33 ± 0.47 (1 ~ 2)	1.27 ± 0.45 (1 ~ 2)	0.615
**ODI**			
Pre-surgery	70.75 ± 10.02	72.63 ± 9.35	0.411
24 h post-surgery	34.27 ± 7.06	35.88 ± 7.45	0.350
6-months post-surgery	23.52 ± 4.45	23.97 ± 4.43	0.672
**Anterior height of diseased vertebrae (mm)**			
Pre-surgery	21.93 ± 4.05	20.95 ± 3.34	0.268
24 h post-surgery	24.72 ± 3.47	23.96 ± 3.36	0.353
6-months post-surgery	24.50 ± 3.42	23.67 ± 3.18	0.290
**Cobb angles(°)**			
Pre-surgery	16.44 ± 9.06	18.01 ± 12.00	0.532
24 h post-surgery	10.76 ± 10.10	12.35 ± 13.53	0.576
6-months post-surgery	10.91 ± 10.05	12.51 ± 13.45	0.571

Cobb’s angles in the PCKP group were 16.44° ± 9.06°, while that of the traditional PKP group was 18.01° ± 12.00° before operation. After the operation, Cobb’s angles decreased to 10.76° ± 10.10° and 12.35° ± 13.53° for PCKP and PKP groups respectively (*P*<0.05). There was no significant difference in Cobb’s angle between the two groups before and after operation (*P*>0.05, Table 2).

### Clinical results

The VAS score and ODI of PCKP and traditional PKP groups were significantly improved 1 day after the operation (*P*<0.05). Six months after surgery, the VAS score and ODI of both groups were also significantly improved compared with that of 24 h after surgery (*P*<0.05). However, there was no difference in VAS score and ODI between PCKP and traditional PKP groups at any time point (Table 2).

### Postoperative complications

**Fig. 5:**
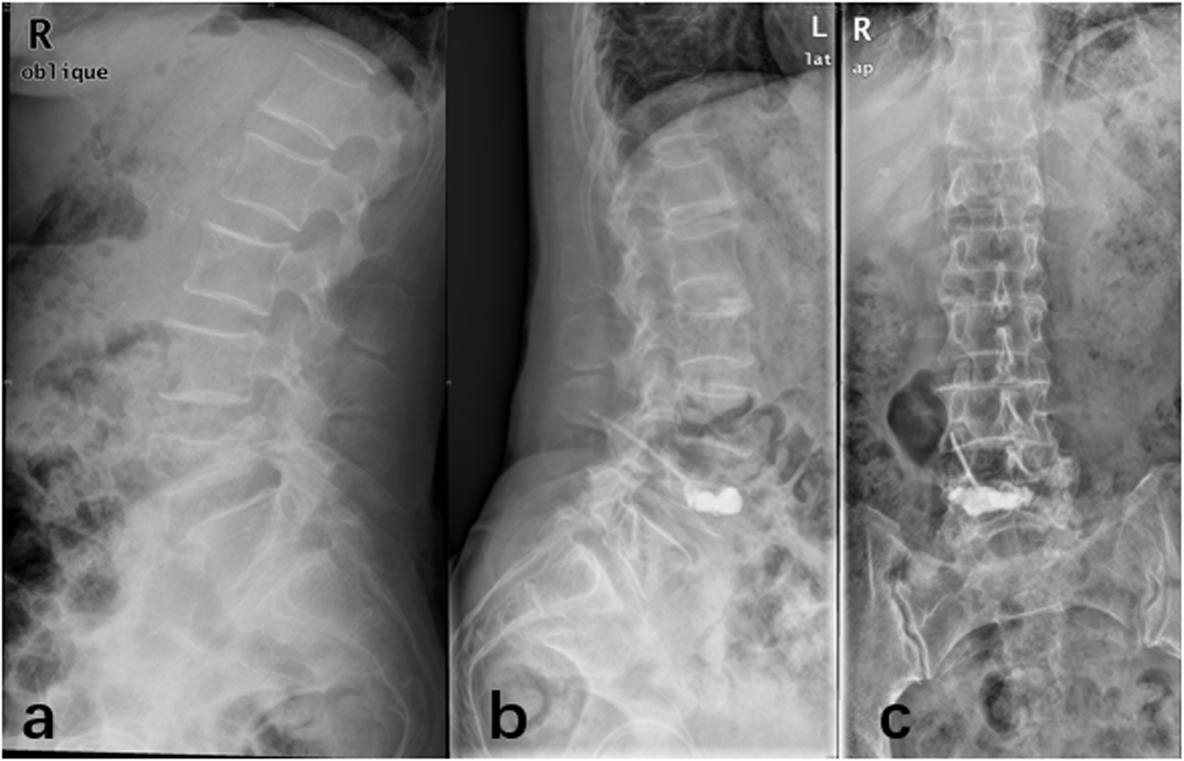
Postoperative bone cement leakage occurred in PCKP group, the patient had no symptoms

In this study, a small amount of bone cement leakage occurred in some patients during surgery. There were 3 patients in the PCKP group (total leakage rate 8.3%, Fig. 5), and 8 patients in the traditional PKP group (total leakage rate 22.2%). Most of the bone cement leakage was characterized by leakage along the fracture line, while 1 case in the traditional PKP group had paravertebral segment intravascular leakage, because the bone cement did not enter the drawing stage and was injected prematurely (Table 3).

**Table 3:** Comparison of postoperative adverse events between the two groups

Classification	PCKP group	PKP group	*P* values
**Bone cement leakage**	3	8	0.101
Intervertebral space	1	3	
Lateral position of vertebrae	1	2	
Anterior position of vertebrae	0	2	
Posterior position of vertebrae	1	0	
Paravertebral segment intravascular	0	1	

In this study, during the perfusion of bone cement, all cases that have used an X-ray real-time dynamic monitor were able to stop the perfusion when the leakage was observed.

## Discussion

Osteoporotic fracture of the vertebral body is very common in the elderly, and traditional treatment requires long-term bed rest, fixation and drug treatment. Due to reduced activity, osteoporosis is further aggravated in patients, and fractures occur repeatedly. Meanwhile, spending a long time in bed leads to bedsore, deep venous thrombosis and other complications [7]. Osteoporotic fractures of vertebral bodies can seriously affect the quality of life of patients and threaten their physical and mental health [8]. Therefore, pain relief, early activity and spinal stabilization are the key points in the treatment of thoracolumbar osteoporotic compression fractures [9]. PKP can reconstruct the vertebral body height, increase the stiffness of vertebral bodies, immediately stabilize the vertebral body, quickly relieve back pain, reduce the complications in bed, improve cardiopulmonary function, improve the quality of life of elderly patients and is currently the preferred treatment of OVCF [10, 11].In recent years many scholars have proposed the use of a unilateral pedicle puncture in PKP [5, 6, 12, 13]. Compared with bilateral PKP, unilateral PKP produced advantages such as a shorter surgery time, smaller dosage of cement, lower risk of cement leakage, and relieved a higher degree of intractable pain at short-term follow-up after surgery [14]. Indeed, unilateral puncture PKP can reduce both the operation time and complications of bilateral punctures [12]. However, it may cause an uneven distribution of bone cement on both sides of the vertebral body, thus resulting in wedge formation of the non-punctured vertebral body, although this is still controversial [15–18]. Therefore, in recent years, increasing attention has been paid to comparative studies on the filling effect of single versus double puncturing cement [4, 19–21].

The unilateral approach has obvious advantages in terms of the operation time, radiation exposure and device cost [6], but it is often necessary to increase the angle of the puncture, thus leading to the penetration of the inner wall of the vertebral pedicle and an increased risk of spinal cord and nerve root injuries. The bilateral approach, on the other hand, has a higher operation time and puncture risk. Some studies [22, 23] have shown that unilateral percutaneous vertebroplasty for OVCF can achieve the same clinical effect as the traditional bilateral approach by grasping the intraoperative insertion angle and using the method of multiple pushing while backing.

The advantage of the unilateral bending vertebroplasty is that it does not need to overemphasize the inclination angle. One only needs to master the basic technique of transpedicle puncture to achieve a symmetrical distribution of bone cement, ensure the continuity of bone cement distribution in the midline area, and provide stronger sagittal plane stress to support spinal injuries [6]. Compared with the traditional direct unilateral approach, which uses “single point and single time” perfusion, the angle type of bone cement injection can not only ensure the uniform distribution of bone cement, but also reduce the injection pressure, thus helping to reduce the leakage rate of bone cement. Performing PKP with a unilateral puncture and a bending angle can lead to a uniform distribution of bone cement on both sides, achieving a similar effect to that of performing a bilateral puncture. Meanwhile, in terms of operation time, puncture risk and X-ray exposure, PCKP also has the same advantages of the unilateral approach.

At present, the results from comparative studies of single and double punctures are not uniform. Tohmeh et al. [17] and Steinmann et al. [18], through *in vitro* mechanical experiments, found that unilateral PKP was effective in reconstructing the stiffness and strength of injured vertebrae and there was no significant difference compared with bilateral puncture. Kim et al. [24] suggested that unilateral puncture PKP was not as effective as a bilateral puncture in restoring vertebral stability. Authors argue that there is an unbalance of the piercing cement filling and a possible mechanical deflection. It has been reported [5] that when unilateral pedicle puncture PKP was performed, bone cement filling was limited to the semi-vertebral body and could restore the axial compression strength of the vertebral body. But under a lateral pressure load, the stiffness of the non-punctured side was significantly lower than that of the punctured side. When the bone cement filling crossed the midline, the stiffness of both sides of the vertebral body can be more evenly enhanced, so as to achieve a balanced enhancement of the vertebral physicochemical performance and reduce the risk of postoperative vertebral physicochemical deflection and wedge fractures on the non-punctured side [25]. In this study, unilateral angle puncture was used for PCKP. When the puncture needle reached the ideal position in the vertebral body, the balloon expanded, and bone cement was dispersed in the front and middle of the vertebral body, which was significantly different from that of PKP after bilateral balloon expansion, in which the bone cement was mainly distributed on the sides of the vertebral body. OVCF were mainly at the collapse of the anterior, middle and endplate of the vertebral body. The volume of bone cement inpoured into the PCKP group was lower than that of the traditional PKP group, but the bone cement in the anterior and middle of the vertebral body was more in line with the biomechanics of the fractured vertebral body.

Bone cement leakage is a serious complication of vertebroplasty. Previous studies [11, 26] have suggested that the fracture of the perivertebral wall or endplate, the pressure of bone cement perfusion and the volume of bone cement perfused are the main causes of bone cement leakage. Throughout this study, we suggest that the direction of injection is also an influencing factor of bone cement leakage. Conventional PKP is required to correct kyphotic deformities of an injured vertebra by injecting bone cement at the point where the puncture needle tip reaches 1/3 of the front of the vertebra. At this point, when the puncture needle is injected with bone cement toward the anterior edge of the vertebra, leakage in the front and side of the bone cement is likely to occur. However, in this study, when the elbow cannula entered the front 1/3 of the vertebral body, the distal end of the cannula was toward the rear side, so the bone cement injection space was large and the bone cement injection pressure was low, which prevented from causing leakage. Indeed, bone cement leakage occurred in only 3 of the 36 vertebral bodies of patients from the PCKP group (8.3%), far lower than that reported in previous literature (about 14.6%) [11]. Intraoperative real-time X-ray fluoroscopy monitoring can reduce the amount of bone cement leakage, thus avoiding pulmonary embolism, spinal canal stenosis, spinal cord compression, nerve injury and other serious complications.There are several limitations to our study. The results of this study may be limited by the relatively short follow-up time (6 months), the relatively small number of participants, and the fact that this was a single-center study. Therefore, the conclusions drawn from this study remain to be validated by larger prospective randomized controlled clinical trials and longer follow-up.

## Conclusion

In conclusion, PCKP has similar short-term effects as traditional bilateral PKP in the treatment of senile osteoporotic compression fractures, while both approaches can obtain satisfactory clinical efficacy, significantly relieve pain and improve the life quality of patients. However, compared with traditional bilateral PKP, PCKP is less invasive, has a shorter operation time, needs fewer X-rays, and has a lower bone cement leakage rate, making PCKP worthy of widespread clinical application.

## Data Availability

We do not wish to share our data due to individual privacy, and according to the policy of our hospital, the data should not be shared to others without permission.

## Abbreviations

PKP: Percutaneous kyphoplasty
PCKP: Percutaneous curved kyphoplasty
OVCFs: Osteoporotic vertebral compression fractures
DI: Oswestry disability index questionnaire
VAS: Visual analogue scale
